# Genetics ignite focus on microglial inflammation in Alzheimer’s disease

**DOI:** 10.1186/s13024-015-0048-1

**Published:** 2015-10-05

**Authors:** Manasi Malik, Ishita Parikh, Jared B. Vasquez, Conor Smith, Leon Tai, Guojun Bu, Mary Jo LaDu, David W. Fardo, G. William Rebeck, Steven Estus

**Affiliations:** Department of Physiology and Sanders-Brown Center on Aging, University of Kentucky, 800 S. Limestone St, Lexington, KY 40536 USA; Department of Anatomy and Cell Biology, University of Illinois, Chicago, IL USA; Department of Neuroscience, Mayo Clinic, Jacksonville, FL USA; Department of Biostatistics and Sanders-Brown Center on Aging, University of Kentucky, Lexington, KY USA; Department of Neuroscience, Georgetown University Medical Center, Washington, DC, USA

**Keywords:** Alzheimer’s disease, Neuroinflammation, Microglia, GWAS, TREM2, CD33, CR1, ABCA7, SHIP1, APOE

## Abstract

In the past five years, a series of large-scale genetic studies have revealed novel risk factors for Alzheimer’s disease (AD). Analyses of these risk factors have focused attention upon the role of immune processes in AD, specifically microglial function. In this review, we discuss interpretation of genetic studies.  We then focus upon six genes implicated by AD genetics that impact microglial function: *TREM2*, *CD33*, *CR1*, *ABCA7*, SHIP1, and *APOE*. We review the literature regarding the biological functions of these six proteins and their putative role in AD pathogenesis. We then present a model for how these factors may interact to modulate microglial function in AD.

## Introduction

Recent large scale genetic studies have identified a set of single nucleotide polymorphisms (SNP)s that are associated with Alzheimer’s disease (AD) risk [1–7]. Several of the genes underlying these SNPs encode proteins relevant to microglial function and inflammation, including TREM2, CD33, CR1, ABCA7 and SHIP1. Prior to these studies, inflammation was well-recognized to occur in AD with a general consensus that anti-inflammatory agents may be helpful. However, the specific aspects of inflammation that were beneficial or detrimental were unclear (reviewed in [8–13]). These recent genetic studies pinpoint specific proteins and pathways in AD risk. When considered along with the pro-inflammatory role of the prototypical AD genetic risk factor, *APOE4*, a model emerges showing that AD risk is reduced by processes that suppress inflammatory cytokine signaling and enhance clearance of debris, including amyloid. In this review, we examine these recent genetic studies and their interpretation, integrate the findings into our understanding of inflammation processes in AD and discuss the potential for their translation into AD preventative and/or therapeutic agents.

The advent of well-powered genome wide association studies (GWAS) has been critical to this advance. Simply put, these studies compare SNP frequencies between AD and non-AD populations. SNPs with significantly different minor allele frequencies between these two populations constitute potential AD risk factors. For many years, researchers performed candidate SNP studies to identify those that associate with AD risk. The resulting studies were, in retrospect, typically underpowered and not reproducible. This difficulty was not restricted to AD research but was rather a common issue in genetic association studies (reviewed in [14]). The large-scale nature of these GWAS and the requirement for independent replication has largely mitigated concerns about power and reproducibility [1–5]. Among the positive findings reported recently, only the SNP associated with CD33 has struggled with reproducibility [4, 7]. There are several interpretations of non-confirmatory studies. First, when considering whether new data are inconsistent with prior findings, we suggest that the analysis include the 95 % confidence intervals for the SNP association with AD in both the “positive” and “negative” study. If the confidence intervals overlap, the studies are not necessarily in disagreement, and perhaps a meta-analysis of the overall dataset would be informative. This is especially appropriate if the follow-up study is underpowered relative to the original study or if the original finding exhibited a winner’s curse bias, thus overestimating the SNP’s correlation with AD risk [15]. A second possible interpretation of inconsistent genetic results stems from the recognition that the SNPs used in GWAS high throughput arrays are selected to be reliably assayed and to provide a wide genomic coverage and high minor allele frequency (>5 %). Hence, GWAS SNPs are rarely functional SNPs. More commonly, a positive SNP from a GWAS is co-inherited (in linkage disequilibrium (LD)) with a functional SNP. If the functional SNP is consistently co-inherited with the GWAS SNP across ethnic groups and races, then the GWAS SNP will be an efficient proxy SNP for the functional SNP and show consistent association with AD across cohorts. However, if the GWAS SNP is not consistently co-inherited with the functional SNP in different populations, the GWAS SNP will show variable association with phenotype. Hence, inconsistent follow-up results need to be carefully interpreted and understanding the functional effects associated with GWAS SNPs is critical to integrate genetic findings into our understanding of AD.

Determining the relevance of AD genetics to AD prevention or therapy requires knowledge of the SNP actions. Association studies estimate the magnitude of the polymorphism's effect on AD risk. To understand the extent to which we need to impact the underlying pathway to affect AD risk, we need to quantify the SNP action on gene function. The critical factor here is that many of the recent SNPs implicated by GWAS have modest odds ratios for AD risk even without adjusting for the winner’s curse [16]. Whether the products of implicated genes represent robust drug targets depends upon the molecular impact of the SNP on protein function. For example, if the genetic variant completely inhibits the protein and yet has only a 0.9 odds ratio, the protein may not be a robust drug target because complete inhibition produces only a modest effect on AD risk. Alternatively, if the genetic variant alters protein function by a modest 10 % and has a 0.9 odds ratio, the protein may represent a robust drug target because a drug could be developed that mimics the protective SNP allele to a greater degree and thus could have a greater effect on AD risk. This hypothesis has underlying caveats, including that the dose-dependence of protein function in AD risk does not reach a premature plateau, that the protein is a suitable drug target, etc. However, *prima facie*, a gene modulated by a SNP that has a modest effect on function and a modest effect on AD risk may represent a robust drug target. A secondary issue to address is the thought process that a genetic-based therapy will apply only to those with the AD-risk allele; this is not necessarily accurate. An ideal intervention will not only mimic the action of a protective allele, it will amplify this effect and therefore may be applicable to individuals regardless of their genotype. That said, the intervention may have more impact on those with the risk allele.

In summary, we present an analysis of the genetics of inflammation and microglia in AD. This analysis focuses upon genes selected by the criteria of (i) the genes contain SNPs implicated in AD risk by compelling genetic studies and (ii) the genes encode proteins that impact microglial activation. These genes include *TREM2, CD33, CR1, ABCA7, SHIP1* and *APOE*. Aspects of AD that are relevant but beyond this focus of inflammation genetics, such as AD neuropathology or amyloid protein precursor metabolism, are reviewed elsewhere [17–20]. Within our analysis of genetics, inflammation, microglia and AD, we will discuss the biology of the relevant protein, the protein’s role in inflammation, and how these proteins may interact to collectively modulate immune function in the AD brain.

## Review

### TREM2

TREM2 (Triggering Receptor Expressed on Myeloid cells 2) is a type 1 transmembrane receptor protein. In mice, TREM2 is expressed in myeloid cells in the brain and appears increased in microglia in the vicinity of plaques in APP mice [21, 22]. Most but not all human brain studies have suggested that TREM2 expression is within microglia [23–26]. TREM2 expression increases with IL-4 exposure [27], suggesting that TREM2 expression may be increased during alternative activation of microglia.

**Fig. 1:**
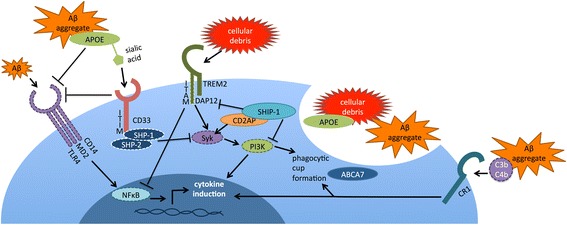
Several interactions have been reported between the AD risk genes involved in inflammation. TREM2 signals through the ITAM of DAP12 to activate microglial phagocytosis; however, TREM2 expression has also been shown to dampen pro-inflammatory cytokine production activated by TLRs. Activated CD33 recruits SHP-1 and SHP-2 to inhibit Syk signaling; CD33 has also been shown to antagonize CD14/TLR4 signaling. Sialylated apoE, which complexes with Aβ, may serve as a CD33 ligand. ApoE appears to dampen TLR4 and TLR2 signaling and inhibit induction of pro-inflammatory cytokines. SHIP1 antagonizes PI3K action by converting PIP3 to PIP2; SHIP1 has also been shown to bind to and antagonize TREM2 /DAP12 signaling in osteoclasts. SHIP1 also complexes with CD2AP, another AD-implicated protein, to inhibit Syk ubiquitination and degradation. CR1 is a C3b/C4b receptor that promotes phagocytosis; complement components have been shown to complex with Aβ. ABCA7 has been localized to phagocytic cups and linked to Aβ clearance, although its mechanism of action is currently unknown. Proteins encoded by genes associated with AD risk by genetics are shown with solid outlines; proteins that mediate these interactions are shown with dashed outlines

TREM2 ligands include anionic lipids and perhaps other unknown elements from apoptotic neurons [28–31]. TREM2 lacks an extended cytosolic domain, signaling through the immunoreceptor tyrosine-based activating motif (ITAM) of its co-receptor, DAP12 [32, 33]. Activated TREM2 stimulates DAP12 through an intramembrane lysine residue, resulting in phosphorylation of the DAP12 ITAM, and activation of the kinase Syk (Fig. 1, [24, 34]). This leads to activation of PI3K, resulting in actin rearrangement and phagocytic cup formation for target engulfment [35, 36]. TREM2-activated phagocytosis occurs without a commensurate activation of cytokine production [37]. Indeed, TREM2 activation actually decreases cytokine production that occurs in response to Toll-like receptor (TLR) activation [27, 38]. The activation of TREM2, Syk and phagocytosis is balanced by activation of phosphatases, most notably SHP-1, SHP-2, and SHIP1 (encoded by the AD-risk gene *INPP5D,* see below). Overall, TREM2 stimulation via apoptotic neuronal fragments or TREM2 antibodies appears to result in activation of microglial phagocytosis with minimal changes in cytokine levels.

Nonsense, missense, and splice site mutations in *TREM2* and its signaling partner DAP12 have been identified as causing Nasu-Hakola disease, a rare, autosomal recessive syndrome marked by early-onset progressive dementia and osteoclast dysfunction resulting in bone cysts [24, 39–42]. In 2012, a genome-wide search for AD risk factors based on the Icelandic population found that a *TREM2* missense mutation, R47H (rs75932628), is a significant AD risk factor [43]. This finding was complemented by a large-scale companion study [44]. The odds ratio associated with R47H was initially estimated at 3–4, sparking great interest into TREM2 as a potentially robust therapeutic target [43, 44]. Subsequent studies have confirmed the association but reduced the magnitude of this odds ratio [45–52]. The R47H variant blunts the TREM2-DAP12 response to anionic lipids [29]; therefore, the mutation may reflect a stage of TREM2 function intermediate between full function and the complete loss of function that is associated with the recessive Nasu-Hakola disease.

While the role of TREM2 in phagocytosis may have important implications for Aβ clearance in AD [53], the R47H variant has also been implicated in Parkinson’s disease and frontotemporal dementia (FTD), neither of which centrally involves amyloid [54]. The Q33X nonsense mutation, which appears to lead to loss of TREM2 function, is also associated with FTD risk [55]. Therefore, TREM2-mediated phagocytosis may also target neuronal debris that accumulates with normal synaptic plasticity and with neuronal loss seen in neurodegenerative disorders. Consistent with this possibility, *TREM2*-transduced macrophages promote clearance of neuronal debris and recovery in an animal model of multiple sclerosis [56].

Based on these findings, many researchers speculate that activators of TREM2 function or expression may represent robust AD preventative and/or therapeutic agents. However, some recent results have called this hypothesis into question. An initial study by Ulrich et al. showed that *TREM2* hemizygosity does not affect the prevalence of cortical plaques, soluble Aβ levels, or production of inflammatory cytokines in APPPS1-21 mice [57]. However, *TREM2* hemizygosity does affect recruitment of myeloid cells, presumed to be microglia, to plaques [57]. A later report by Wang et al. involving 5xFAD TREM2 knockout and hemizygous mice showed similar results, with greatly reduced microglial clustering around plaques in *TREM2*-depleted mice [29]. As many researchers had initially hypothesized from the genetic findings, this study found that *TREM2*-deficient mice develop more plaques and higher soluble and insoluble Aβ levels. However, this effect did not appear to be due to differences in Aβ uptake or metabolism [29]. Instead, *TREM2* deletion resulted in decreased microgliosis and microglial survival, at least in part due to decreased response to CSF-1 [29].

In agreement with these two studies, an independent group found that in both 5xFAD and APP-PS1 mouse models, *TREM2* knockout mice had negligible immune cells clustering around plaques [58]. Surprisingly, Jay et al. found that *TREM2* deletion led to decreased plaque levels in the hippocampus and unchanged plaque levels in the cortex [58]. These amyloid results appear incongruent with the study by Wang et al. and with genetic findings that seem to point to a protective role for TREM2 in neurodegenerative disease [29, 55]. The reason for these discrepancies is unclear, although one variable is that the two groups use different *TREM2* knockout strains: Jay et al. use a strain that lacks exons 2–4, which encode the ligand binding domain through the cytosolic domain, while Wang et al. use a strain that lacks exons 3–4, which encode a portion of the TREM2 transmembrane and cytosolic sequence [19] and could produce soluble TREM2.

Even more intriguingly, when Jay et al. examined the “microglial” myeloid cells surrounding plaques in TREM2-positive mice, they found that they had higher CD45 expression than normal microglia, suggesting that they might in fact be bone-marrow derived monocytes infiltrating from the periphery [58]. The authors proposed that the effects of TREM2 in AD are mediated by infiltrating monocytes, rather than microglia: in fact, they report that TREM2 expression in resident microglia in the brain is undetectable by immunohistochemistry. While awaiting further supporting evidence, this model offers insight into therapeutic strategies for AD: pharmacologic agents may not need to cross the blood–brain barrier to be effective but may be able to act on peripheral monocytes that then invade the brain. Overall, studies on TREM2 have focused unparalleled research attention on this inflammation pathway, implicating microglial activation in general and phagocytosis in particular as critical for healthy CNS aging.

### CD33

CD33 is a type 1 transmembrane protein and member of the sialic acid-binding immunoglobulin-like lectin (SIGLEC) family of receptors (reviewed in [59]). In the brain, CD33 is expressed largely in microglial cells [60, 61]. CD33 ligands appear to include sialylated cell surface proteins acting in *cis* as well as other sialyated agents. Many have suggested that CD33 (like other inhibitor SIGLECs) functions to limit immune activation in response to “self” macromolecules that bear a “self-associated molecular pattern” of sialylation (reviewed in [62]). Indeed, amyloid plaques may be “hidden” from microglia because the plaque vicinity is rich in sialylated agents including apoE, apoJ and gangliosides that stimulate CD33 and thereby inhibit microglia [63–65].

Sialic acid binding activates CD33, resulting in phosphorylation of the CD33 immunoreceptor tyrosine-based inhibitory motif (ITIM) domains and activation of the SHP-1 and SHP-2 tyrosine phosphatases [66, 67]. These phosphatases act on multiple substrates, including Syk, to inhibit immune activation [68, 69]. Hence, CD33 activation leads to increased SHP-1 and SHP-2 activity that antagonizes Syk, inhibiting ITAM-signaling proteins, possibly including TREM2/DAP12 (Fig. 1, [70, 71]). Consistent with this possibility, long-term CD33 inhibition (by antibodies or siRNA) induces production of cytokines such as IL1β, TNFα, and IL-8 [72].

A polymorphism in the *CD33* proximal promoter was associated with AD risk in several, although not all genome wide studies [4, 6, 7, 73]. Recently, we and others sought to identify the mechanism whereby rs3865444 modulates CD33 to alter AD risk [60, 61, 74–76]. CD33 expression in the brain is modestly increased in AD and decreased with the minor, AD-protective rs3865444A allele [60, 61, 76]. Increased CD33 expression was associated with reduced Aβ phagocytosis [60, 76]. This is consistent with findings that CD33 activation by cell surface sialic acids in *cis* leads to reduced cellular activation [72, 77], as well as a report suggesting that CD33 negatively regulates CD14, an Aβ receptor [78–80].

Pursuing these molecular actions further, we found that the rs3865444A minor allele was associated with a robust increase in the proportion of *CD33* isoforms that lacked exon 2 (*D2-CD33*) or retained intron 1; the former change results in a translated CD33 protein that lacks its ligand-binding IgV domain, while the latter change results in a frameshift and premature stop codon [61, 74, 75, 81, 82]. Additionally, DNA sequencing established that rs12459419, a SNP within *CD33* exon 2, was in near- perfect LD with rs3865444. *In vitro* minigene splicing studies demonstrated that rs12459419 is a functional SNP that modulates the efficiency of exon 2 splicing [61]. Since D2-CD33 lacks the predicted sialic acid binding domain,* D2-CD33* likely encodes a non-functional protein ([81, 82], reviewed in [59]); consistent with this possibility, Aβ42 uptake is inhibited when BV-2 cells were transfected with *CD33 *but not when cells were transfected with *CD33 *lacking the IgV domain [60]. Overall, these results provide a genetic basis to support a model wherein TREM2 and CD33 act in opposing directions to modulate tyrosine phosphorylation and, thereby, microglial activation and AD risk.

The utility of CD33 as a target for AD prevention or therapy is an active research area. Of particular interest, the mRNA splicing studies show that each minor allele of the AD-associated SNP results in a 24 % decrease per allele in *CD33 *mRNA including exon 2 and lacking intron 1 [75]. Hence, an AD odds ratio of ~0.89 correlated with a ~24 % reduction in RNA encoding functional CD33. This suggests that more robust CD33 inhibition may reduce AD risk further. This suggestion is supported further by studies with *CD33* deficient mice; these mice develop normally and yet have reduced amyloid accumulation [60, 83], suggesting that CD33 inhibition in humans may be safe and robustly reduce AD risk. Recently, we showed the CD33 antibody Lintuzumab, which has been used safely in human acute myeloid leukemia trials, downregulates cell surface CD33 up to 80 % in PMA-differentiated U937 cells in vitro [75]. Based on linear regression of the allelic dose dependence for *CD33* isoforms and AD risk, this 80 % reduction is calculated to reduce the AD odds ratio to 0.68, consistent with the possibility that this level of inhibition could be clinically meaningful.

### INPP5D (SHIP1)

The gene *INPP5D* encodes phosphatidylinositol-3,4,5-trisphosphate-5-phosphatase 1, also known as SHIP1 (SH2-containing inositol 5′ - phosphatase). Since SHIP1 is expressed in macrophage cell lines [84], SHIP1 may be expressed in microglia in the brain. SHIP1 is a phosphatase that hydrolyzes PIP3 to PIP2 on the cytosolic side of plasma membrane, counteracting PI3K induced pathways [85]. The SHIP1 amino-terminal region contains an SH2 domain that binds phosphorylated tyrosine residues on ITIM- or ITAM-containing target proteins [85–90]. SHIP1 has been shown to inhibit monocyte activation and phagocytosis [68, 84, 91–95], in part by transducing inhibitory signaling of FcγRIIB and other ITIM-containing proteins. In dendritic cells, SHIP1 complexes with the AD-relevant protein CD2AP to inhibit the ubiquitination of pro-inflammatory proteins Syk and FcγRIIa [96]. SHIP1 also reduces NF-κB activation, which has been shown to activate BACE1 expression in activated astrocytes [97, 98]. Perhaps most relevant to AD, SHIP1 inhibits TREM2 signaling through DAP12 in osteoclasts, dysfunction of which is another hallmark of Nasu-Hakola disease [99]. Interestingly, SHIP1 does not appear to inhibit the ITIM of CD33 [66, 100, 101].

An INPP5D polymorphism, rs35349669, has recently been associated with AD risk [4]. SHIP1 transcription is  initiated at multiple start sites; initiation at an internal site results in production of a protein lacking the SH2 domain [102]. Rs35349669 is near this internal transcription start site, suggesting that this SNP may modulate production of a SHIP1 transcript lacking the SH2 domain. Understanding the actions of the AD-associated SNP rs35349669 may be critical to understanding the role of SHIP1 in AD. Overall, SHIP1 appears to transduce inhibitory signaling of some ITIM-containing proteins and to inhibit signaling of ITAM-containing proteins, such as DAP12. Hence, SHIP1 is anti-inflammatory and anti-phagocytic.

### CR1

CR1 (Complement Receptor 1) regulates the complement system, a division of the body’s innate immune response that orchestrates phagocytosis and lysis of cells bearing foreign antigens. CR1 is expressed on some leukocytes in the periphery, as well as the choroid plexus, microglia, and neurons in the brain [103, 104]. CR1 acts as both a positive and negative regulator of the complement pathways by binding to the C3b/C4b peptides, stimulating (i) opsonization and clearance of immune complexes and (ii) destabilization of the C3 and C5 convertases, preventing further complement activation.

The complement pathway has been associated with AD since the 1982 when complement factors were found in amyloid plaques [105]. The complement protein C1q was subsequently shown to bind Aβ, leading to complement activation and inflammation [106]. Addition of C1q to solubilized Aβ1-42 promotes Aβ aggregate formation [107]. Subsequent studies found that complement pathway proteins are upregulated in AD brain [108], and that C3b binding to Aβ leads to inflammation and neuronal lysis (reviewed in [109]). However, some studies have found that the complement cascade can have neuroprotective effects: the complement protein C5a has been shown to activate MAPK, protecting neurons and reducing hippocampal lesions in mouse models [109]. Recent results suggest that CR1 has a mixture of neuroprotective and neurodegenerative effects in AD: antagonizing CR1 prevents Aß phagocytosis by primary rat microglia, but also blocks microglial production of superoxide species and the pro-inflammatory cytokines TNFα and IL-1β. Blockage of CR1 also prevents neuronal death when neurons are treated with microglial conditioned media [110]. These data suggest that CR1 activation may be beneficial to clear Aβ at early disease stages but exacerbate inflammation once amyloid deposits have appeared.

SNPs in *CR1* have been associated with AD risk in GWAS since 2009 [1, 4, 6, 7]. The search for functional SNPs that mediate this association with AD has revealed two candidates. First, a rare coding SNP rs4844609 (S1610T) was associated with several indices of AD pathology [111]; however, these initial associations were not replicated in an independent cohort [112]. Second, AD-associated SNPs from GWAS such as rs4844610 were associated with a copy number variant (CNV) that modulates the production of two CR1 isoforms: a larger isoform designated CR1-S (slow migrating on gel electrophoresis) and a smaller isoform designated CR1-F (fast migrating) [113]. The larger CR1-S isoform, which has a 15 % frequency and associates with increased AD risk, contains two copies of low copy repeat 1 and therefore encodes more C3b/C4b binding sites than the smaller CR1-F [113]. This CNV is a better predictor of AD risk than the GWAS-implicated SNP rs4844610, suggesting that *CR1* genetics modulate AD risk through the functional CR1 CNV [104]. The action of CR1-S in AD is still unclear: one possibility is that since CR1-S encodes more C3b/C4b binding sites, CR1-S leads to increased complement activation and inflammation and thereby increases AD risk [104, 113]. However, studies have also shown that CR1-S carriers have lower overall CR1 protein expression: therefore, AD risk might be conferred through lower CR1 expression leading to decreased complement activation and impaired clearance of Aβ [104, 114]. Hence, whether CR1 and complement activation are beneficial or deleterious for AD is currently unclear and requires further study.

### ABCA7

ABCA7 is a member of the ATP-binding cassette superfamily of transporters that is expressed in the periphery in the spleen, thymus, and bone marrow, as well as in microglia in the brain [115, 116]. ABCA7 was initially thought to modulate lipid homeostasis and was found to transport phospholipids across inner and outer plasma membrane leaflets [117–119]. However, the *C. elegans* ABCA7 homolog, ced-7, is responsible for apoptotic cell engulfment [120]. In fact, ABCA7 has been localized to the phagocytic cups of activated microglia, although the underlying mechanism is not fully understood [120]. Consistent with the possibility that ABCA7 may modulate lipid transport and thereby contribute to phagocytosis, Rong et al. observed that inflammation modulates membrane phospholipid composition, thus affecting the function of membrane proteins [121]. Moreover, Kim et al. showed ABCA7 deficiency resulted in increased Aβ deposition, suggesting a decrease in phagocytic clearance [122]. Taken together, these findings support the possibility that ABCA7 reduces AD risk by contributing to phagocytic cup formation and Aβ clearance (Fig. 1).

Several *ABCA7* SNPs, including rs3764650, have been associated with AD in initial and replicative GWAS [4, 7]. The mechanisms underlying this association are unclear. We recently reported that the AD-protective rs3764650 allele was associated with increased ABCA7 expression [123], leading us to hypothesize that the SNP-associated increase in *ABCA7* expression protects from AD. We also saw an increase in *ABCA7* expression in AD brains that we attributed to an increase in inflammation in AD, noting that ABCA7 expression is increased as monocytes differentiate into macrophages and, interestingly, by LDL [115]. A recent study of an Icelandic population showed that rare loss-of-function variants in *ABCA7* confer an increased risk of AD, with a combined odds ratio of 2.1. In replication populations, this striking association held true with an odds ratio of 1.7. One of these variants, rs200538373, promotes retention of a short intronic sequence after exon 41, resulting in a premature stop codon [124]. Hence, an apparent loss of ABCA7 function increases AD risk while increased *ABCA7* expression is associated with reduced AD risk.

### APOE

While these recent genetic studies have focused new attention upon genes that are critical to neuroinflammation, *APOE* polymorphisms have also been suggested to differentially affect inflammation. Of the three common *APOE* alleles, *APOE4* raises AD risk and reduces age of AD onset, while *APOE2* lowers AD risk and increases age of onset [125]. In the CNS, *APOE* is primarily expressed by astrocytes [126], as well as by microglia and ependymal cells and, under certain conditions of neurotoxicity, by neurons [127]. ApoE functions in the transport of cholesterol (reviewed in [128]), and is important for the redistribution of lipids within the CNS, including delivery of cholesterol and phospholipids to neurons.

In addition to its function in lipid transport, apoE is an anti-inflammatory agent. Evidence supporting this perspective includes that apoE deficiency exacerbates neuroinflammation in several rodent injury models including ischemia [129], experimental autoimmune encephalomyelitis [130], traumatic brain injury [131], and induced neuroinflammation [132]. Moreover, apoE deficiency was associated with a reduced clearance of neuronal debris in a model of entorhinal cortex lesion, suggesting that apoE contributes to the clearance of cholesterol-rich neuronal breakdown products [133]. Conversely, an apoE mimetic peptide decreased damage in traumatic brain injury [134], increased axonal regeneration after peripheral nerve injury [135], and decreased lesion volume after focal ischemia [136].

*C*ompared to *APOE3*, *APOE4* is associated with a reduced ability to suppress inflammatory stimuli both *in vivo* and *in vitro* (reviewed in [137]). In models comparing *APOE*-knockout (KO) mice to human *APOE*-targeted replacement (TR) mice, *APOE4* appears to represent a loss of positive function rather than a gain of negative function. For example, in *APOE*-TR mice treated with intracerebroventricular (ICV) injections of lipopolysaccharide (LPS), levels of activated microglia, astrocytes, invading T-cells and cytokines (IL-1β, and TNFα), and synaptic protein loss were greater in *APOE*-KO > *APOE4*-TR > *APOE3*-TR > *APOE2*-TR [138]. Similarly, in the periphery, proinflammatory stimuli induced an increase in IL-1β release in *APOE*-KO > *APOE4*-TR > *APOE3*-TR > *APOE2*-TR [139]. Peripheral LPS injection also induces higher TNFα levels in *APOE4*-TR compared to *APOE3*-TR mice [140, 141], while TNFα, IL-6, and IL-1β are greater in *APOE*-KO mice compared to wild type mice [132]. Although the literature is sparse on *APOE* isoform-specific effects on Aß-induced neuroinflammation, evidence supports that *APOE4* modulates Aß-induced neuroinflammation *in vivo*. E4FAD mice (*APOE*-TR mice crossed with 5xFAD mice) [142] exhibit greater microgliosis and astrogliosis around cortical Aβ deposits compared to E3FAD [143]. Multiplex analysis of mRNA levels for neuroinflammatory markers in the cortex of EFAD mice at 6 and 8 months revealed that in 6 month E4FAD mice, select markers related to TLR4 signaling are higher, while IL-4R and related markers are lower compared to E3FAD mice; these age- and *APOE*-dependent effects suggest an *APOE*3-specific adaptive response lacking with *APOE4* [137].

These *in vivo* findings that apoE promotes an anti-inflammatory state and that apoE4 is a less effective anti-inflammatory agent than apoE3 are recapitulated *in vitro*. In response to LPS stimulation, TNFα and IL-6 are upregulated in *APOE*-KO glial cells compared to wild type [132], while nitric oxide levels are increased in *APOE*-KO but not *APOE3*-TR mixed glial cultures [144]. In primary microglia, an apoE-mimetic peptide inhibits LPS-induced JNK activation through interactions with the LDL receptor family members [145, 146]. *APOE* deletion has also been shown to upregulate TLR4 and TLR2 and enhance TLR signaling [147, 148]. Compared to apoE3, apoE4 increases cytokine production in both LPS-treated peripheral immune cells [132, 133] and LPS- and Aß-treated mixed glial cultures [137, 149, 150]. LPS and oligomeric Aβ-induced TNFα secretion are inhibited by TLR4 antagonists in mixed glial cultures, consistent with APOE isoform specific effects on TLR4 signaling *in vivo* [137, 149, 150]. *APOE4* alleles also dose-dependently increase nitric oxide production in microglial cultures [141].

Overall, the distinction that *APOE4* represents a loss of positive function in neuroinflammation rather than a gain of negative function is important with respect to development of AD treatment strategies, suggesting that *APOE4* carriers would benefit from strategies designed to correct the structure/function of apoE4 rather than eliminate apoE4.

### Proposed Model Integrating Genetics, Neuroinflammation and AD Risk

We propose a testable model wherein each AD genetic risk factor considered here is integrated into a coherent model wherein AD risk is modulated by immune activation. We depict activation as defined by promoting phagocytosis and cytokine production (Fig. 1). We recognize that microglial cytokine production has diverse effects in AD; these effects have been reviewed elsewhere [151].

Hence, we suggest the following:

### TREM2

Neuronal debris and perhaps other lipid-rich ligands activate TREM2, which promotes microglial phagocytosis through the ITAM domain of its co-factor, DAP12 and the downstream effector, Syk [28]. We hypothesize that enhanced TREM2 function reduces AD risk, although as discussed above, this hypothesis is not supported by all current data [29, 57, 58].

### CD33

Sialic acid-rich areas, such as the vicinity of plaques, stimulate CD33 signaling, leading to activation of the CD33 ITIM, which, in turn activates SHP-1 to inhibit microglial activation, particularly TREM2 signaling via Syk [70, 71]. ApoE is an abundant sialylated protein in the vicinity of plaques and hence could contribute to this action [63–65]. The AD-protective SNP allele reduces the proportion of CD33 encoding functional CD33 and thereby effectively inhibits CD33 to promote microglial activation [60, 61, 74–76].

### INPP5D (SHIP1)

The SH2-containing SHIP1 isoform moves from the cytosol to the cell surface to bind the phosphorylated ITAM of DAP12 to inhibit TREM2 signaling [99]. SHIP1 also antagonizes the action of PI3K, an important mediator of phagocytosis [95]. We hypothesize that the risk allele of the AD-associated SNP will increase expression of SHIP1 or a SHIP1 isoform.

### CR1

This receptor is stimulated by C3b and C4b, which may bind to Aβ to promote inflammation and/or phagocytosis. The CR1 GWAS signal is most likely due to a CNV which modulates the proportion of the two CR1 isoforms: the shorter CR1-F and longer CR1-S [113]. The CR1-S isoform, which increases AD risk, has more ligand-binding sites but also leads to decreased overall expression; therefore, it is unclear whether CR1 activation is beneficial or deleterious for AD risk [104, 113, 114].

### ABCA7

This phagocytic cup protein is critical to phagocytosis of substrates such as apoptotic cells [120]. The AD-risky allele of rs3764650 was associated with modestly decreased ABCA7 expression [7, 123]. More strikingly, rare nonsense SNPs were associated with a more robust AD odds ratio [124]. Overall, decreased ABCA7 is hypothesized to reduce phagocytosis and thereby increase AD risk.

### APOE

This protein promotes an anti-inflammatory state, with apoE4 being less effective than apoE3, which is less effective than apoE2. Hence, we propose that the AD risk associated with apoE4 is due, at least in part, to increased pro-inflammatory microglial activation with reduced phagocytosis. Conversely, apoE2 reduces AD risk relative to apoE3 by promoting an anti-inflammatory state, perhaps with increased phagocytosis. These apoE allelic actions could occur in parallel with other apoE allele-dependent mechanisms that also modulate AD, including differential ability to serve as an Aß chaperone (reviewed in [128]).

**Fig. 2:**
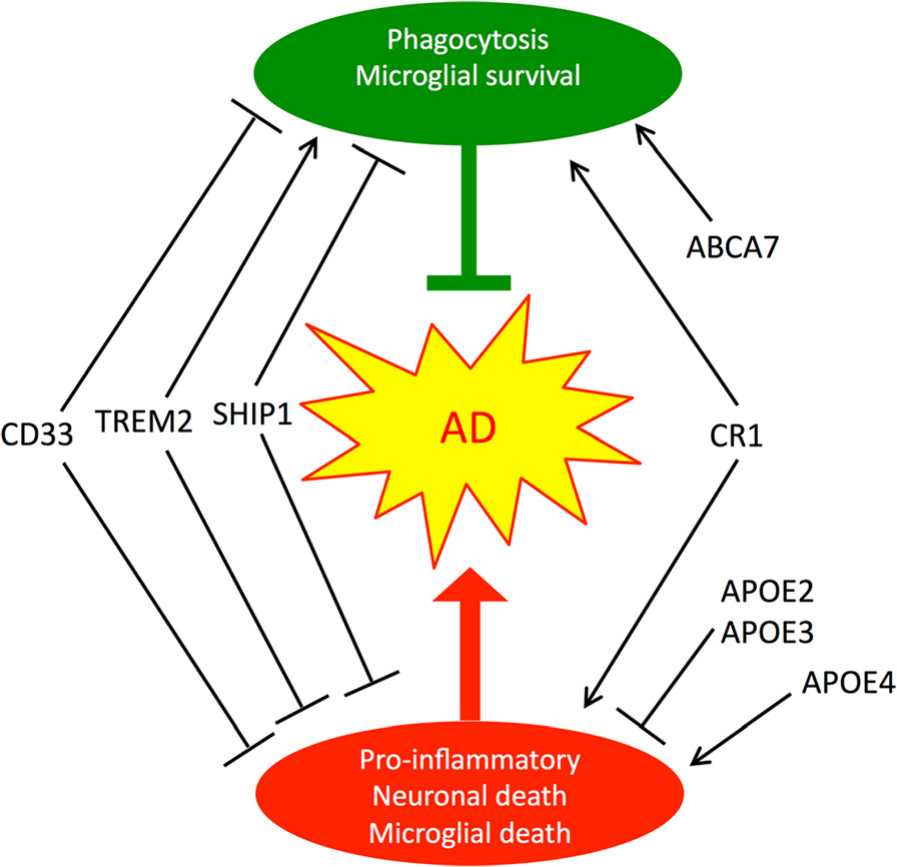
Microglial activation can be neuroprotective and/or neurotoxic; the actions of AD risk proteins modulate these effects. The normal actions of CD33 and SHIP1 (encoded by *INPP5D*) appear to antagonize both forms of microglial activation, while CR1 action appears to promote both Aβ phagocytosis and the production of neurotoxic pro-inflammatory cytokines such as TNF. TREM2 appears to promote phagocytosis while dampening pro-inflammatory cytokine production. ABCA7 helps to mediate phagocytosis. *APOE2* and *APOE3* are anti-inflammatory, while *APOE4* promotes inflammation and neurotoxicity

In aggregate, we propose that microglial activation state reflects a homeostatic balance between proteins like CD33 and SHIP1 that inhibit all microglial activation, proteins like TREM2 that promote phagocytic activity without inflammatory cytokines, and proteins like CR1 that are non-specific immune activators. We would also group apoE4 in the last category, relative to the immunosuppressive activities of apoE3 and, progressively, apoE2 (Fig. 2).

## Conclusions

Genetics has certainly ignited interest in the role of inflammation as a modulator of AD pathogenesis and risk. Along with this interest has come renewed appreciation that immune activation represents a “double-edged sword” in that activation can be neuroprotective by promoting phagocytosis and clearance of cellular debris and Aβ, but also neurotoxic by promoting pro-inflammatory cytokine production, oxidative stress, and neuronal death. While the actions of some AD-relevant agents, such as TREM2 polarize towards a protective phenotype, other agents, such as CD33, CR1, and SHIP1 (*INPP5D*) seem to modulate both “types” of inflammation concurrently, suggesting that these two phenotypes may not in fact be distinct or separable [152].

Since the actions of several AD-associated SNPs are still unknown, further research into SNP effects on gene expression and protein function is needed to gain clarity. However, even where there is widespread agreement in the field as to the effect of a genetic variant, results from different variants tell different tales. For example, CD33 inactivation and *APOE4* seem to have similar effects on TLR activation and pro-inflammatory cytokine induction [72, 78] but genetic data tell us that CD33 inactivation is AD-protective while *APOE4* is of course risky [141, 147]. This suggests that an agent that generally modulates even one facet of microglial activation, such as TLR function or phagocytosis, may not be useful as an AD therapeutic: currently, the field has not yet determined with certainty that any particular microglial function is “good” or “bad” for AD risk. However, we *can* currently be confident that a genetic risk factor discovered in unbiased, large-scale human studies is protective or risky for AD. This is in part why genetic risk factors offer such great promise as therapeutic targets and research tools, and why it is so critical to understand their effects.

## Abbreviations

SNP: Single nucleotide polymorphism
AD: Alzheimer’s disease
GWAS: Genome wide association studies
LD: Linkage disequilibrium
TREM2: Triggering receptor expressed on myeloid cells 2
ITAM: Immunoreceptor tyrosine-based activating motif
TLR: Toll-like receptor
FTD: Frontotemporal dementia
SIGLEC: Sialic acid-binding immunoglobulin-like lectin
ITIM: Immunoreceptor tyrosine-based inhibitory motif
SHIP1: SH2-containing inositol 5′ - phosphatase
CR1: Complement Receptor 1
CNV: Copy number variant
KO: Knockout
TR: Targeted replacement
ICV: Intracerebroventricular
LPS: Lipopolysaccharide
